# High incidence of MYC and BCL2 abnormalities in mantle cell lymphoma, although only MYC abnormality predicts poor survival

**DOI:** 10.18632/oncotarget.5705

**Published:** 2015-10-23

**Authors:** Shuhua Yi, Dehui Zou, Chengwen Li, Shizhen Zhong, Weiwei Chen, Zengjun Li, Wenjie Xiong, Wei Liu, Enbin Liu, Rui Cui, Kun Ru, Peihong Zhang, Yan Xu, Gang An, Rui Lv, Junyuan Qi, Jianxiang Wang, Tao Cheng, Lugui Qiu

**Affiliations:** ^1^ State Key Laboratory of Experimental Hematology, Institute of Hematology and Blood Disease Hospital, Chinese Academy of Medical Sciences and Peking Union Medical College, Tianjin, China; ^2^ Department of Hematology, Tianjin First Center Hospital, Tianjin, China

**Keywords:** mantle cell lymphoma, MYC, BCL2, P53, prognosis

## Abstract

The incidence and prognostic role of MYC and BCL2 rearrangements in mature B-cell lymphomas have been extensively studied, except the infrequent mantle cell lymphoma (MCL). Here, we analyzed the MYC and BCL2 abnormalities and other cytogenetic aberrations by fluorescence *in situ* hybridization (FISH) in 50 MCL patients with bone marrow involvement. Eighteen patients (36.0%) had MYC gains and/or amplifications, and twelve patients (24.0%) had BCL2 gains and/or amplifications. Among the 18 patients with MYC abnormality, four had simultaneous MYC translocations, but no BCL2 translocation was detected among patients with BCL2 abnormality. Only two patients (4.0%) had both MYC and BCL2 abnormalities. The patients with a MYC abnormality had a significantly higher tumor burden, a higher percentage of medium/high risk MIPI group and genomic instability compared to those without this abnormality. However, no significant difference was observed between patients with or without a BCL2 abnormality in terms of clinical and cytogenetic factors. Patients with a MYC abnormality had poorer progress-free survival (PFS) (9.0 vs. 48.0 months, *p* = .000) and overall survival (OS) (12.0 vs. 94.5 months, *p* = .000), but the presence of a BCL2 abnormality did not significantly influence either PFS or OS. In multivariate analysis, the MYC abnormality was the independent adverse factor for both PFS and OS, and intensive chemotherapy did not improve the outcome of these patients. Thus, the presence of a MYC but not BCL2 abnormality predicted the poor survival of MCL patients, and a new treatment strategy should be developed for these patients.

## INTRODUCTION

Recently, increasing attention has been given to a subset of mature B-cell lymphomas with both MYC and BCL2 rearrangements, which have been defined as “double hit” lymphomas (DH lymphomas) because of their aggressive clinical course and resistance to conventional chemotherapy [1]. DH lymphoma accounts for approximately 10% of diffuse large B cell lymphoma (DLBCL) cases and has poor survival even when treated with rituximab combined chemotherapy [2]. However, other lymphomas with MYC and BCL2 rearrangements have received less attention. For example, no previous study has systematically described the incidence and evaluated the prognostic role of a double hit with MYC and BCL2 rearrangements in mantle cell lymphoma (MCL). In this study, we will describe the incidence of MYC and BCL2 abnormalities detected by fluorescence *in situ* hybridization (FISH) and discuss the prognostic role of cytogenetic aberrations in MCL.

## RESULTS

### Clinical characteristics

As shown in Table 1, the median age of the 50 patients was 55.5 years (range 33–91); there were 38 male patients (76%). All of the patients had bone marrow involvement at diagnosis that was identified by flow cytometry and bone marrow biopsy. The indolent MCL was excluded by the short clinical course and aggressive medical history. Eighteen patients (36%) had B symptoms and 36 patients (72%) had splenomegaly at diagnosis. The median white blood cell (WBC) was 44.73 × 10^9^/L (range 2.63–193.78), and the median β2 microglobulin (MG) was 4.45 mg/L (range from 1.95 to 12.7). Based on the MCL international prognostic index (MIPI) system, 26 patients (52%) were classified as high-risk, and 12 patients each (24%) were classified as medium- and low-risk.

**Table 1:** The clinical characteristics of 50 MCL patients

Characteristics	
Median age(range, year)	55.5(33–91)
Sex (male)	38(76%)
Median WBC(×10^9^/L)	44.37(2.63–193.78)
Median β2-MG(mg/L)	4.55(1.95–12.7)
MIPI	
Low risk	12(24%)
Medium risk	12(24%)
High risk	26(52%)
B symptom	18 (36%)
splenomegaly	36(72%)
hepatomegaly	3(6%)

Abbreviation: WBC, white blood cell; MG, microglobulin; MIPI, MCL international prognostic index.

### Cytogenetic aberrations

All of the patients were CCND1/IGH positive according to FISH, as the diagnosis required. Thirty-eight patients (76%) had at least one of the second cytogenetic aberrations. The incidences of the second cytogenetic aberrations were as follows: eighteen patients had a 13q deletion (36.0%); nine patients had an ATM deletion (18.0%); seventeen patients had a TP53 deletion (34.0%); eighteen patients had a MYC abnormality (36.0%); and twelve had a BCL2 abnormality (24.0%). Among the eighteen patients with a MYC abnormality, all of them had a MYC gains and/or amplifications and only 4 patients had translocation signals detected by the MYC dual color break-apart rearrangement probe. These patients did not exhibit a translocation with the IGH gene, as there was no abnormal signal with the IGH/MYC dual fusion probes. However, all of the BCL2 abnormalities were only gains and/or amplifications, without a translocation signal with the BCL2 dual color break apart probe or the IGH/BCL2 dual fusion translocation probe. Only 2 patients had a MYC and BCL2 abnormality simultaneously, whereas 11 patients had both a MYC abnormality and a TP53 deletion. Because the gain and amplification always come out concurrently in an individual, we did not discriminate these two situations.

In order to validate the above results, we tested MYC and BCL2 abnormalities in five MCL cell lines: Z138, JVM-13, Granta-519, MAVER1, JEKO-1, which were given kindly by professor John Chan in University of Nebraska Medical Center. Two cell lines have MYC amplification (MAVER1 and JEKO1); Two with MYC gain (Granta519 and JVM-13) and Z-138 with translocation. In aspect of BCL2, no translocation was observed and JVM-13 had normal BCL2, while other four cell lines having BCL2 amplification. These results are according with above phenomenon that gain/amplification of MYC and/or BCL2 are more frequent than translocation in MCL.

Then, we compared the clinical characteristics and cytogenetic aberrations between patients with a MYC or BCL2 abnormality and those who did not have these abnormalities. As shown in Table 2, there was no significant difference between patients with or without a BCL2 abnormality in terms of clinical and cytogenetic factors except for in the case of their total cytogenetic aberrations. However, most of the clinical factors and all of the secondary cytogenetic aberrations were significantly different in patients with a MYC abnormality compared to those patients without this abnormality.

**Table 2:** The comparison of the clinical and biological characteristics between MYC/BCL2 abnormality or not

Clinical characterstics	MYC+	MYC−	*p* value	BCL2+	BCL2−	*p* value
*N*	18	32		12	38	
Median age (year)	55.0(33–71)	55.0(46–99)	.606	52.0(33–91)	55.0(42–79)	.481
Median WBC(×10^9^/L)	35.43(2.63–168.89)	12.96(3.06–193.78)	.052	14.32(3.91–84.88)	22.14(2.63–193.78)	.413
Median β2MG(mg/L)	6.67(2.09–12.7)	3.76(1.95–6.76)	.009	3.76(3.02–5.63)	4.63(1.95–12.7)	.429
MIPI			.036			.129
Low risk	1(5.6%)	11(34.4%)		7(58.3%)	31(81.6%)	
Medium/High risk	17(94.4%)	21(65.6%)		5(41.7%)	7(18.4%)	
B symptom	9(50%)	9(28.1%)	.122	5(41.7%)	13(34.2%)	.693
splenomegaly	3(16.7%)	0	.017	0	3(7.9)	.315
hepatomegaly	14(77.8%)	22(68.8%)	.495	8(66.7%)	28(73.7%)	.637
Del(13q)	11(61.1%)	7(21.9%)	.006	2(16.7%)	16(42.1%)	.170
Del(11q)	6(33.3%)	3(9.4%)	.034	1(8.3%)	8(21.1%)	.425
Del(17p)	11(61.1%)	6(18.8%)	.002	2(16.7%)	15(39.5%)	.181
BCL2 abnormality	2(11.1%)	10(31.3%)	.170	MYC+ 2(16.7%)	MYC+ 16(42.1%)	.170
Total abberation	18(100%)	20(62.5%)	.002	12(100%)	26(68.4%)	.047

Abbreviation: WBC, white blood cell; MG, microglobulin; MIPI, MCL international prognostic index.

### Survival and prognosis

**Figure 1:**
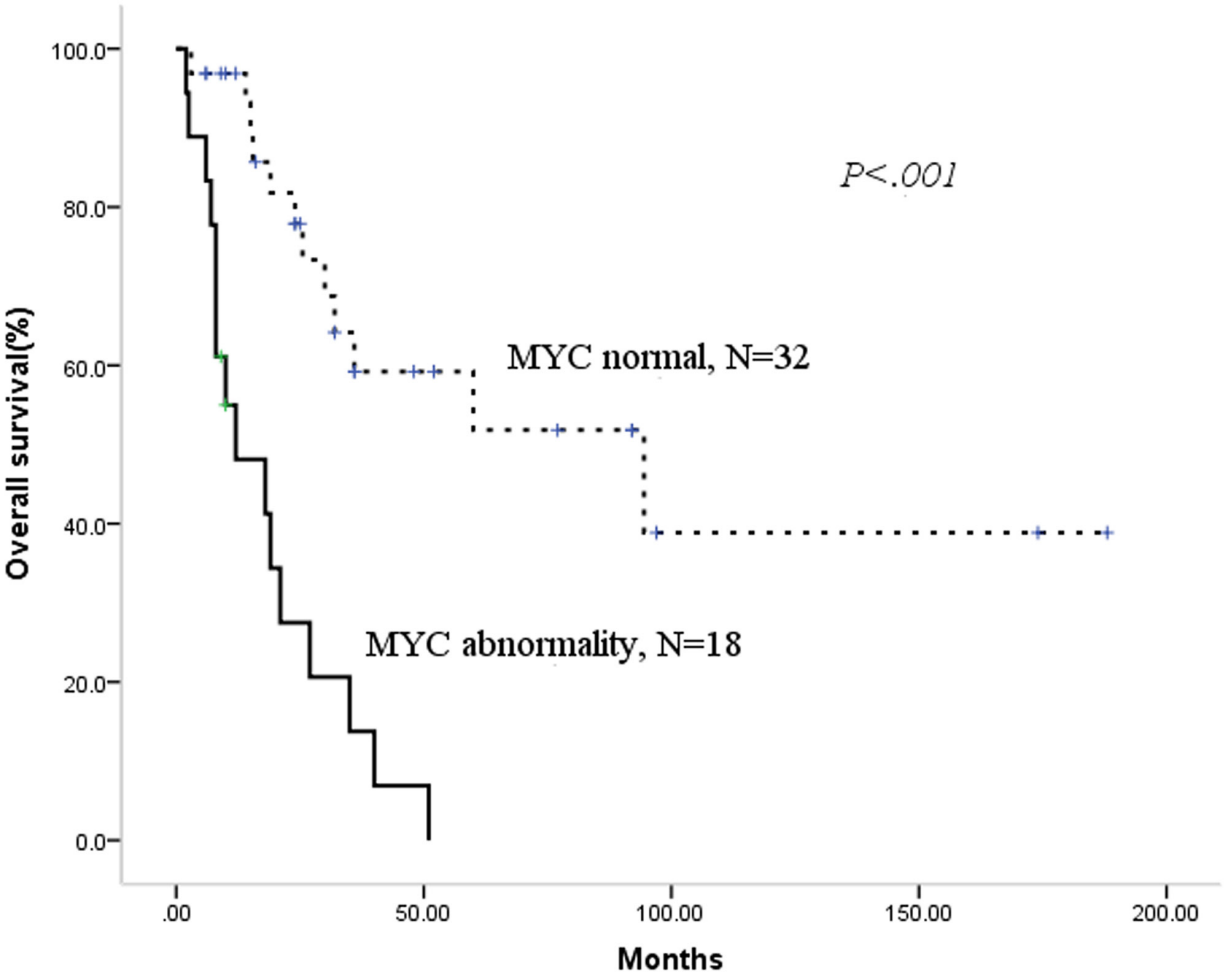
Patients with MYC abnormality have adverse progress-free survival

**Figure 2:**
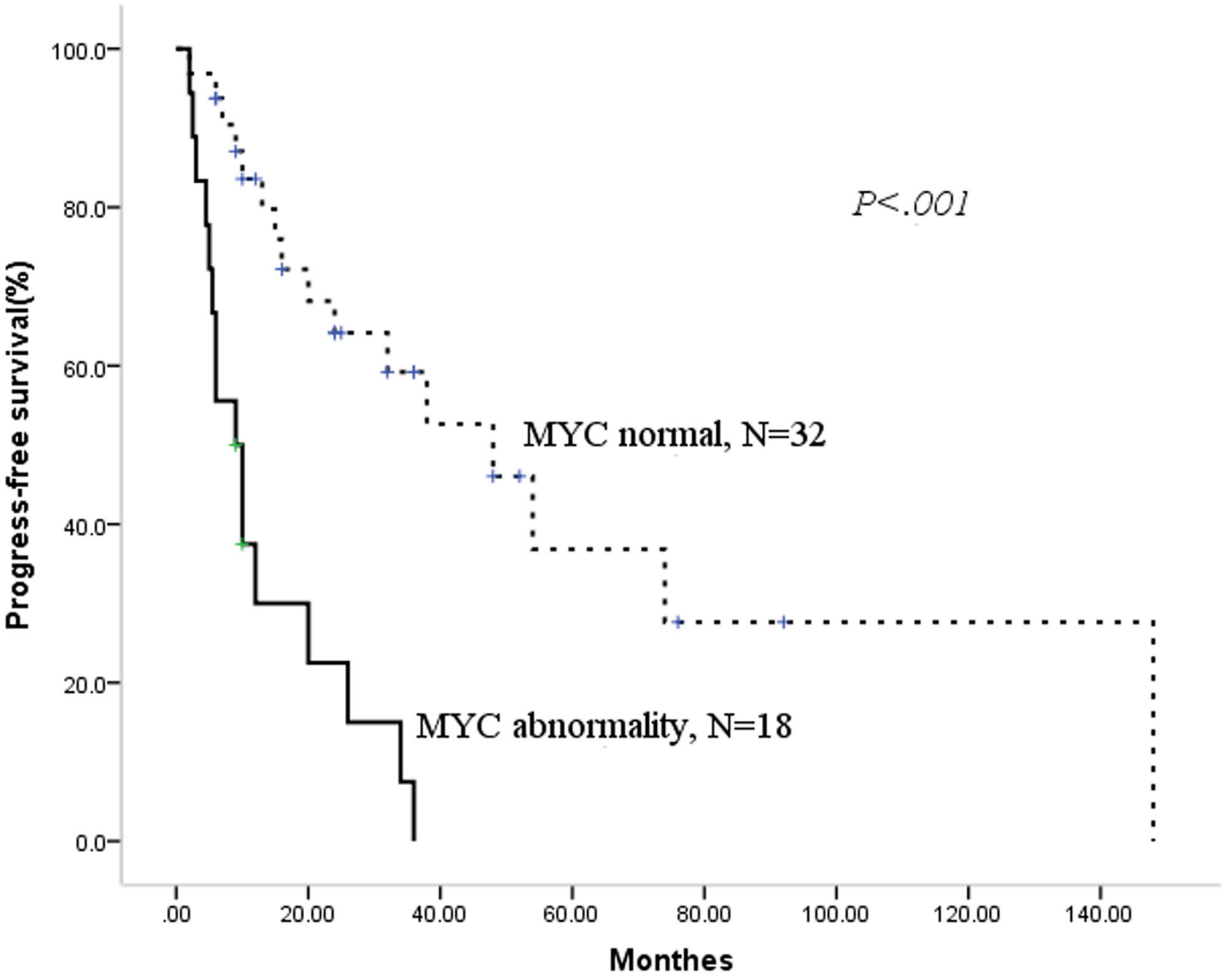
Patients with MYC abnormality have adverse overall survival

During the median follow-up period of 22.5 months (range 2–188.0), 28 patients had died. The median overall survival (OS) for the entire cohort of patients was 32.0 months (95%CI 20.8–43.2), with median progress-free survival (PFS) of 24 months (95% CI 6.9–41.1). Then, we analyzed the prognostic factors for this population. As shown in Table 3, we identified using the Kaplan-Meier method that MIPI high-risk status, a 13q or 17p deletion, MYC abnormality and any cytogenetic aberrations had an adverse prognostic effect on PFS. In addition, the deletion of 13q or 17p, MYC abnormality and receiving CHOP/CHOP-like ± R chemotherapy had an adverse prognostic effect on OS. BCL2 abnormality did not significantly influence neither PFS nor OS. Then, we constructed a Cox regression models for PFS and OS using the positive factors in the univariate analysis. Deletions of 17p and MYC abnormality were the two independent factors for PFS and OS, as shown in Table 4 and Figures 1 and 2.

**Table 3:** The univariate analysis of prognostic factors for PFS and OS

Factors	*N*	PFS	*p* value	OS	*p* value
Sex			.359		.492
Mal	38	26.0(6.4–45.6)		35.0(21.0–49.0)	
femal	12	24.0(0–86.4)		32.0(16.3–47.7)	
B symptom			.664		.451
Yes	18	20.0(8.8–31.2)		21.0(14.1–27.9)	
No	32	32.0(16.6–47.4)		36.0(25.1–46.9)	
MIPI			.034		.097
Low risk	12	148.0(not reached)		Not reached	
Medium risk	12	32.0(19.3–44.7)		36.0(13.4–58.6)	
High risk	26	10.0(6.6–13.4)		19.0(10.4–27.6)	
Del 13q			.003		.012
Yes	18	10.0(8.6–11.4)		19.0(7.7–30.3)	
No	32	38.0(10.5–65.5)		94.5(2.4–186.6)	
Del 11q			.091		.060
Yes	9	6.0(4.5–7.5)		10.0(4.2–15.8)	
No	41	32.0(14.2–49.8)		32.0(16.6–47.4)	
Del 17p			.000		.000
Yes	17	9.0(5.5–12.5)		14.0(4.0–24.0)	
No	33	38.0(17.5–58.5)		60.0(5.3–114.7)	
MYC abnormality			.000		.000
Yes	32	9.0(3.9–14.1)		12.0(0–25.9)	
No	18	48.0(26.5–69.5)		94.5(15.3–173.6)	
BCL2 abnormality			.283		.466
Yes	12	24.0(11.9–36.1)		30.0(13.2–46.8)	
No	38	26.0(0–54.2)		32.0(0–73.2)	
Any cytogenetic aberrations*			.023		.008
No	12	16.0(5.4–26.6)		Not reached	
Yes	38	48.0(26.6–69.4)		25.5(15.9–35.1)	
Chemotherapy			.094		.040
HyperCVAD/MA ± R	27	38.0(0–90.7)		Not reached	
CHOP/CHOP-like ± R	23	20.0(8.8–31.2)		32.0(12.1–51.9)	

Abbreviation: MIPI, MCL international prognostic index; Del, deletion.

* indicate patients with either del 13q/11q/17p,or BCL2/MYC aberration.

**Table 4:** Multivariate analysis for PFS and OS using Cox regression model

		RR	95% CI	*P* value
PFS	MIPI	1.6	0.9–3.0	.135
	Deletion 13q	1.1	0.5–2.8	.761
	Deletions 17p	2.5	1.1–5.9	.039
	MYC abnormality	3.1	1.2–7.8	.020
	Any cytogenetic aberration*	0.9	0.3–3.5	.923
OS	Deletions 13q	0.9	0.4–2.3	.829
	Deletions 17p	2.4	1.02– 5.8	.045
	MYC abnormality	3.21	1.15–8.96	.026
	Any cytogenetic aberration*	2.5	0.5–12.3	.257
	CHOP/CHOP-like ± R Treatment	.831	0.5–2.5	.831

Abbreviation: MIPI, MCL international prognostic index; PFS, progress-free survival; OS, overall survival.

* indicate patients with either del 13q/11q/17p,or BCL2/MYC aberration.

As the chemotherapeutic regimens significantly influence the survival (shown in Table 3), we want to know whether the regimens containing high-dose cytarabine could overcome the adverse prognosis of MYC abnormality or del 17p. Firstly, we validate the prognostic role of MYC abnormality and del 17p in 27 patients treated with HyperCVAD ± R/MA ± R. There were 8 patients with MYC abnormality and del 17p respectively. Patients with MYC abnormality had significantly shorter median PFS (5.5 vs. 74.0 months, *p* = .003) and OS (10 months vs. not reached, *p* < .001) compared with those without. Del 17p also played a adverse role in this treatment background for both PFS (5.5 vs. 74.0 months, *p* = .001) and OS (10 months vs. not reached, *p* = .001). Then, we focused on the therapy of patients with a MYC abnormality. Five patients received R-Hyper CVAD/R-MA alternative chemotherapy, three received the Hyper CVAD/MA regimen, and the other ten patients were treated with CHOP/CHOP-like ± R regimen. There was no advantage with regard to PFS (median PFS 5.5 vs.10.0 months, *p* = .231) or OS (median OS 10.0 vs. 12.0 months, *p* = .416) for the 8 patients treated with intensive chemotherapy compared with the 10 patients who received non-intensive chemotherapy. The chemotherapeutic model also did not change the survival of patients with del 17p. Among 17 patients with del 17p, eight patients were treated with HyperCVAD ± R/MA ± R while nine with CHOP/CHOP-like ± R. The median PFS (5.5 vs.12.0 months, *p* = .415) and OS (10.0 vs.14.0 months, *p* = .521) for these two regimens were similar.

## DISCUSSION

The term ‘DH lymphoma’ was initially used to describe mature-B-cell lymphomas with a chromosomal breakpoint affecting the MYC locus in combination with another recurrent breakpoint, such as BCL2, BCL6, BCL3 or CCND1. The most common type of double-hit occurs with a MYC/8q24 and BCL2/18q21 breakpoint [1]. However, DHL is not a diagnosis recognized in the WHO classification system, and the DHL only refers to DLBCL, B-cell lymphoma unclassifiable with features intermediate between DLBCL and Burkitt lymphoma(BCLU), follicular lymphoma and even lymphoblastic lymphoma in the literature. Thus, the definition of DH lymphoma is controversial. Swerdlow ST [2] proposed that DH lymphomas should be restricted to either DLBCL, NOS or BCLU cases that cannot be better classified as a more specific type of lymphoma. A mantle cell lymphoma with a MYC and CCND1 translocation should still be diagnosed as a mantle cell lymphoma even if the MYC rearrangement has additional clinicopathologic implications. The incidence of MYC/BCL2 DHL is 2–12% in DLBCL and 32–78% in BCLU [2] and is associated with aggressive clinical characteristics and poor survival. The concurrent presence of a MYC and CCND1 rearrangement is rare, accounting for approximately 10% of the patients with double hit and triple hit lymphomas in the Mitelman database [1]. However, the incidence of MYC/BCL2 double rearrangements in MCL had not been reported and it is not known what role MYC/BCL2 rearrangement has in MCL.

Setoodeh R et al [3] reported four MCL cases with MYC abnormality and summarized the characteristics of 26 MCL cases with secondary cytogenetic abnormalities involving the MYC gene in the literature. This publication was a comprehensive report on MYC abnormalities in MCL. Among the reported four cases, three cases exhibited a MYC gene translocation and one demonstrated MYC amplification [3]. Additionally, for the 26 cases with MYC abnormality in the literature, 15 had a MYC translocation and 11 had an additional 8q24 or MYC amplification as analyzed by FISH or conventional karyotype [3]. However, in our series all the 18 patients had a MYC gain/amplification and only four patients had an accompanying MYC translocation, which was definitely different from the literature [3–5].

In the literatures, patients with a MYC abnormity always had extensive bone marrow involvement or leukemic presentation [3, 6–8]. In this study, all of the 50 patients had extensive bone marrow and/or peripheral blood involvement. We compared the clinical and other secondary cytogenetic aberrations between patients with and without a MYC abnormality. There was a higher median WBC, higher percentage of patients belonging to the medium-high MIPI risk group and high tumor burden with higher β2-MG for the MYC abnormality group. The MYC abnormality group also had higher genomic instability as more patients in this group had secondary cytogenetic aberrations. It is well known that MYC dysregulation has the ability to affect gene regulation, microRNA expression profiles, large genomic amplifications and ultimately initiates a dynamic process of genomic instability that is linked to tumor initiation [9]. So, cases of MCL with a MYC abnormality represent a relatively unique group with highly aggressive clinical and biological behavior.

MYC abnormality had been reported to be associated with high proliferation index in MCL [3, 6, 7]. High Ki67 index is also a strong prognosis predicator in MCL with a generally accepted cut-off 30% [10, 11]. We had detected Ki67 index in 13 patients with lymph node biopsy, with three patients had Ki67 index more than 30% (data not shown). Among these 13 patients, four had MYC abnormality with 2 having Ki67 more than 30% (2/4, 50%), while nine patients had no MYC aberration with only one patient having high Ki67 index (1/9, 11.1%). So patients with MYC abnormality may have high Ki67 index but without statistic significance (*p* = .203) in this study, which may attribute to the limited size of available patients.

Intensive chemotherapy, such as the HyperCVAD/MA ± R regimen, can improve the survival of younger MCL patients, and this strategy has been established as the standard treatment for this population [12]. Then, we determined if the HyperCVAD/MA ± R regimen could improve the survival of patients with a MYC abnormality. Unfortunately, this regimen had no impact on the survival of these patients. MYC has been reported to amplify B cell receptor (BCR) signaling, which plays a important role in the pathogenesis of MCL, and increases its own levels via upregulation of miR-17-92 and subsequent targeting immunoreceptor tyrosine inhibitory motif (ITIM) proteins. Consistent with the propensity of ITIM proteins to recruit phosphatases, expression of MYC sustain the BCR signaling pathway by phosphorylation of it's downstream such as spleen tyrosine kinase (SYK) and the B-cell linker protein (BLNK) [13]. So BCR signaling pathway inhibitors may have efficiency in patients with MYC abnormality. Actually, BTK inhibitor ibrutinib has shown amazing results for relapsed and/or refractory MCL [14, 15]. Other new drug, such as lenalidomide [16], also aim to function as a high efficiency treatment for patients with relapsed or refractory MCL. A new strategy targeting MYC-dependent tumors is also under development [17] and may improve the outcome of these patients in the future.

Unlike studies of MYC, there have been no studies about BCL2 abnormalities in MCL. Therefore, we first demonstrated a high incidence of BCL2 amplification in MCL with extensive BMI, and no BCL2 translocations were identified. Some studies have reported that 11–26% patients demonstrate a gain of 18q11-q23, where the BCL2 gene is located, detected by comparative genomic hybridization [18]. This incidence rate is in accordance with our results. Unlike other aggressive B-cell lymphomas, BCL2 translocation may not occur in MCL, which indicates that BCL2 and CCND1 translocations might be mutually exclusive. As high levels of Bcl-2 may indicate that downstream apoptotic pathways are still functional and can be a marker of chemosensitivity and favorable prognosis in certain cancers [19], it is not surprising BCL2 abnormality did not have a prognostic role for MCL in this study.

MCL has some similarities with chronic lymphocytic leukemia (CLL), such as a mature lymphocyte morphology and expression of CD5. The cytogenetic aberrations that frequently occur in CLL have been evaluated in MCL [20]. Unlike in CLL, the prognostic role of del 17p and 11q is controversial. Some studies have found that losses of 17p13 and 11q22-q23 were not associated with poorer outcome [20–22], but other researchers have observed a significant correlation between overall survival and the loss of 11q22-q23 and 17p13 [6, 23, 24]. The loss of 13q14 is an independent factor for overall survival in many studies [20, 22, 23], which is significantly different from CLL in which the loss of 13q14 is considered a good marker for survival. In our study, del 13q, del11q and del 17p all influence survival of MCL patients, but only del17p was an independent adverse prognostic factor for these patients.

As we know, t(11;14) translocation and cyclin D1 overexpression is thought to be the primary event in the pathogenesis of the tumour. But cyclin D1 is not enough to develop spontaneous lymphomas in transgenic mice. And low numbers of cells carrying the t(11;14) translocation have been found in the blood of 1–2% of healthy individuals without any evidence of disease [18]. So second cytogenetic hit is required for the lymphomagenesis of MCL. The MYC abnormality may act as the accomplice to the tumour transformation and aggressive behavior. MYC had been reported to cooperate with cyclin D1 to develop B cell lymphoma in transgenic mice [25, 26]. In aspect of the aggressive clinical outcome, MYC abnormality may be one of the important second cytogenetic event for the evolution of MCL. MCL is one of the malignant lymphoid neoplasms with the highest level of genomic instability. The DNA damage response pathway alterations may constitute another important pathogenetic mechanism in this lymphoma [18]. Deletion of ATM and/or p53 is the main alternation of this pathway. So this may partially explain why patients with p53 or ATM deletion had poor survival.

In conclusion, there was a high incidence of MYC and BCL2 gain and/or amplification in MCL patients with bone marrow involvement. MYC abnormality is the independent adverse factor for PFS and OS, and BCL2 amplification had no significant influence on survival in MCL patients. Intensive chemotherapy such as HyperCVAD/MA ± R did not improve the survival of patients with a MYC abnormality, and a new treatment strategy should be developed. This is a preliminary conclusion and should be validated in multicentric studies based on larger cohort patients under unique chemotherapy.

## PATIENTS AND METHODS

### Patients

During the period from July 2003 through November 2012, 50 MCL patients with bone marrow involvement (BMI) were diagnosed at the Institute of Hematology and Blood Disease Hospital, Chinese Academy of Medical Sciences and Peking Union Medical College (CAMS & PUMC). All patients enrolled informed consent in accordance with requirements of the Declaration of Helsinki, and the research project was approved by the Institutional Review Boards. The essential medical records captured in this study included basic demographics (age, gender), performance status, Ann Arbor stage, presence of extra nodal sites, chromosome karyotype, bone marrow biology and peripheral blood morphology, hematological parameters, and lactate dehydrogenase (LDH) levels, as well as treatment and outcomes. Histologic specimens were reviewed by hematopathologists, including two of the authors, according to the WHO classification system [27]. All bone marrow tissues evaluated in this study were assessed at the time of initial staging. This study was approved by the Ethics Committee of CAMS & PUMC.

### Fluorescence *in situ* hybridization (FISH)

FISH analysis was performed on the samples for conventional karyotype studies. The DNA probe ‘panels’ included probes for 13q14.3 (LSI D13S25 and RB-1), 14q32 (LSI IGHC/IGHV), 17p13 (LSI p53), 11q22 (LSI ATM), LSI BCL2 (18q32) and MYC (8q24) Dual Color, Break Apart Rearrangement Probe, LSI IGH/BCL2 Dual Color, Dual Fusion Translocation Probe and LSI IGH/MYC/CEP 8 Tri-Color Dual Fusion Probes and LSI CCND1/IGH Dual Color, Dual Fusion Translocation Probe. All probes were purchased from Vysis (USA). Sample preparations and hybridizations were conducted following the manufacturer's recommendations and as previously described [28]. Signal screening was carried out on at least 200 cells with well delineated signals. The cut-off for positive values (mean of normal control + 3SD) determined from samples of ten cytogenetically normal people were: 6.5% for the deletion of Rb1, ATM and P53; 4.5% for IgH translocation, LSI BCL2 and MYC Dual Color, Break Apart Rearrangement Probe; and 3.2% for LSI IGH/BCL2 and CCND1/IGH Dual Color, Dual Fusion Translocation Probe and LSI IGH/MYC/CEP 8 Tri-Color Dual Fusion Probes. Gains were defined as three copies of the gene studied, whereas at least four copies were considered as amplifications [29].

### Treatment

All of the 50 patients had received at least 2 cycles of chemotherapy with 80% patients completing the presupposed chemotherapeutic cycles. Twenty-seven patients received HyperCVAD ± R (cyclophosphamide, vincristine, doxorubicin and dexamethasone ± rituximab) alternating with MA (methotrexate and cytarabine) ± R, whereas twenty-three patients received CHOP (cyclophosphamide, vincristine, doxorubicin and dexamethasone ± rituximab) or CHOP-like ± R regimens. Twenty-six patients received rituximab combined chemotherapy. Of the 27 patients treated with HyperCVAD ± R alternating with MA ± R, the median chemotherapeutic cycle is 6 (2–9), 22 patients (81%) had completed at least 6 cycles. For the 23 patients in the CHOP/CHOP-like ± R group, the median cycle is 8 (2–10) with 18 patients(78%) had received at least 6 chemotherapeutic cycles.

### Survival and statistical analysis

Overall survival (OS) was measured as the interval between the date of treatment and the date of death or last follow-up. Progression-free survival (PFS) was measured as the interval between the date of treatment and the date of death from any cause or disease progression. Fisher's exact test or the chi-square test was used to determine statistically significant differences between the clinical characteristics of the two groups. Survival curves were constructed using the Kaplan-Meier method, and prognostic features were evaluated by univariate analysis (log-rank test). The effects of potential prognostic variables on survival were assessed according to the Cox regression method. *P* values < .05 were considered statistically significant. All calculations were performed using the SPSS statistical software package (Version 13.0).
